# Supplementary material for: Discrimination of the Lame Limb in Horses Using a Machine Learning Method (Support Vector Machine) Based on Asymmetry Indices Measured by the EQUISYM System
Source: Sensors (Basel). 2025 Feb 12;25(4):1095. doi: 10.3390/s25041095 (PMC11858852; doi:10.3390/s25041095)
Supplement: Supplementary file 1 [file sensors-25-01095-s001.zip › sensors-3394709-supplementary.pdf]

Table S1. Mean value (± standard deviation) of the variables of the head (_H), withers (_W) and pelvis (_P) used in the model for each group: sound horses, right forelimb (RF) lameness, left forelimb (LF) lameness, right hindlimb (RH) lameness and left hindlimb (LH) lameness.

	Straight Line				
	**Sound**	**RF**	**LF**	**RH**	**LH**
**AsI-up_H** (%)	0.47 (± 28.54)	49.60 (± 34.51)	-50.18 (± 34.06)	18.95 (± 32.52)	-27.60 (± 35.32)
**AsI-up_W** (%)	1.84 (± 8.77)	25.54 (± 18.69)	-23.67 (± 18.52)	-7.74 (± 12.71)	16.99 (± 16.19)
**AsI-up_P** (%)	4.67 (± 11.39)	-7.28 (± 15.42)	12.62 (± 16.81)	28.21 (± 16.99)	-34.51 (± 20.23)
**AsI-down_H** (%)	2.82 (± 26.25)	26.62 (± 32.10)	-33.28 (± 36.16)	20.04 (± 25.08)	-16.43 (± 30.46)
**AsI-down_W** (%)	-0.09 (± 11.21)	4.53 (± 15.99)	-10.38 (± 22.27)	-3.20 (± 8.22)	0.62 (± 14.66)
**AsI-down_P** (%)	3.97 (± 7.61)	6.62 (± 10.97)	-0.42 (± 13.56)	-0.34 (± 12.89)	9.92 (± 14.48)
**AsI-max_H** (%)	0.83 (± 12.60)	19.21 (± 21.77)	-14.36 (± 26.44)	0.14 (± 16.93)	-5.67 (± 21.97)
**AsI-max_W** (%)	1.50 (± 7.16)	11.11 (± 9.93)	-6.21 (± 13.18)	-2.14 (± 6.74)	7.94 (± 11.18)
**AsI-max_P** (%)	-0.78 (± 6.10)	-8.03 (± 11.16)	5.89 (± 12.08)	13.60 (± 9.91)	-22.56 (± 12.75)
**AsI-min_H** (%)	0.26 (± 21.17)	36.54 (± 30.44)	-44.22 (± 31.12)	18.68 (± 25.85)	-24.99 (± 29.50)
**AsI-min_W** (%)	0.34 (± 7.96)	14.43 (± 14.12)	-17.46 (± 18.00)	-5.61 (± 8.40)	9.06 (± 11.44)
**AsI-min_P** (%)	5.45 (± 7.63)	0.75 (± 7.96)	6.73 (± 10.01)	14.60 (± 11.43)	-11.94 (± 12.62)
**AsI-retraction_W** (%)	4.68 (± 8.49)	-5.94 (± 13.16)	11.40 (± 16.91)	2.43 (± 12.74)	3.15 (± 13.26)
**AsI-retraction_P** (%)	7.67 (± 13.80)	7.60 (± 12.63)	4.96 (± 13.83)	-16.65 (± 15.14)	28.12 (± 24.58)
**AsI-Tmax_H** (%)	1.44 (± 12.47)	21.90 (± 20.46)	-25.30 (± 23.80)	9.83 (± 13.41)	-12.35 (± 16.77)
**AsI-Tmax_W** (%)	0.28 (± 1.72)	5.57 (± 5.03)	-4.96 (± 7.31)	-0.31 (± 3.08)	2.97 (± 3.34)
**AsI-Tmax_P** (%)	0.32 (± 3.37)	1.85 (± 3.11)	-0.91 (± 3.78)	-3.58 (± 5.01)	8.48 (± 6.80)
**ERz_H** (%)	72.91 (± 13.59)	56.72 (± 18.30)	53.21 (± 18.47)	69.41 (± 13.23)	63.81 (± 17.38)
**ERz_W** (%)	90.33 (± 3.89)	79.53 (± 11.42)	76.97 (± 12.78)	88.51 (± 5.41)	82.57 (± 8.34)
**ERz_P** (%)	89.14 (± 5.59)	85.67 (± 7.66)	83.93 (± 8.61)	77.20 (± 9.39)	72.32 (± 12.91)
**Δϕ _H** (°)	28.11 (± 96.06)	-75.01 (± 82.68)	64.05 (± 50.20)	-74.90 (± 65.87)	59.39 (± 69.82)
**Δϕ _W** (°)	31.03 (± 92.65)	-41.11 (± 95.45)	59.96 (± 40.99)	42.78 (± 59.07)	-16.21 (± 89.35)
**Δϕ _P** (°)	65.24 (± 70.73)	50.84 (± 96.78)	46.53 (± 60.87)	36.90 (± 41.72)	-20.44 (± 90.79)
**Stride frequency** (Hz)	0.76 (± 0.05)	0.74 (± 0.05)	0.74 (± 0.05)	0.74 (± 0.04)	0.72 (± 0.05)
**ROM_H** (%)	4.18 (± 1.26)	2.82 (± 1.62)	2.62 (± 1.58)	3.21 (± 1.02)	2.78 (± 1.35)
**ROM_W** (%)	6.63 (± 1.17)	5.28 (± 1.58)	5.09 (± 1.56)	6.01 (± 0.99)	5.34 (± 1.21)
**ROM_P** (%)	7.28 (± 1.16)	6.81 (± 1.33)	6.47 (± 1.54)	5.95 (± 1.31)	5.34 (± 1.63)
